# Supplementary material for: Genetic interaction profiles of regulatory kinases differ between environmental conditions and cellular states
Source: Mol Syst Biol. 2020 May 25;16(5):e9167. doi: 10.15252/msb.20199167 (PMC7247079; doi:10.15252/msb.20199167)
Supplement: Supplementary file 1 — Appendix [file MSB-16-e9167-s001.pdf]

Appendix

Genetic interaction profiles of regulatory kinases differ between environmental conditions and cellular states

Siyu Sun[1,2], Anastasia Baryshnikova[3], Nathan Brandt[1,2] and David Gresham[1,2*]

1. Center for Genomics and Systems Biology;
2. Department of Biology, New York University, New York, 10003, USA.;
3. Calico Life Science LLC. South San Francisco, 94080, USA.;
*Correspondence: dgresham@nyu.edu

Table of Contents

1. Appendix FigureS1 ········· 2
2. Appendix FigureS2 ········· 3
3. Appendix FigureS3 ········· 4
4. Appendix FigureS4 ········· 5
5. Appendix FigureS5 ········· 6
6. Appendix FigureS6 ········· 7,8
7. Appendix FigureS7 ········· 9

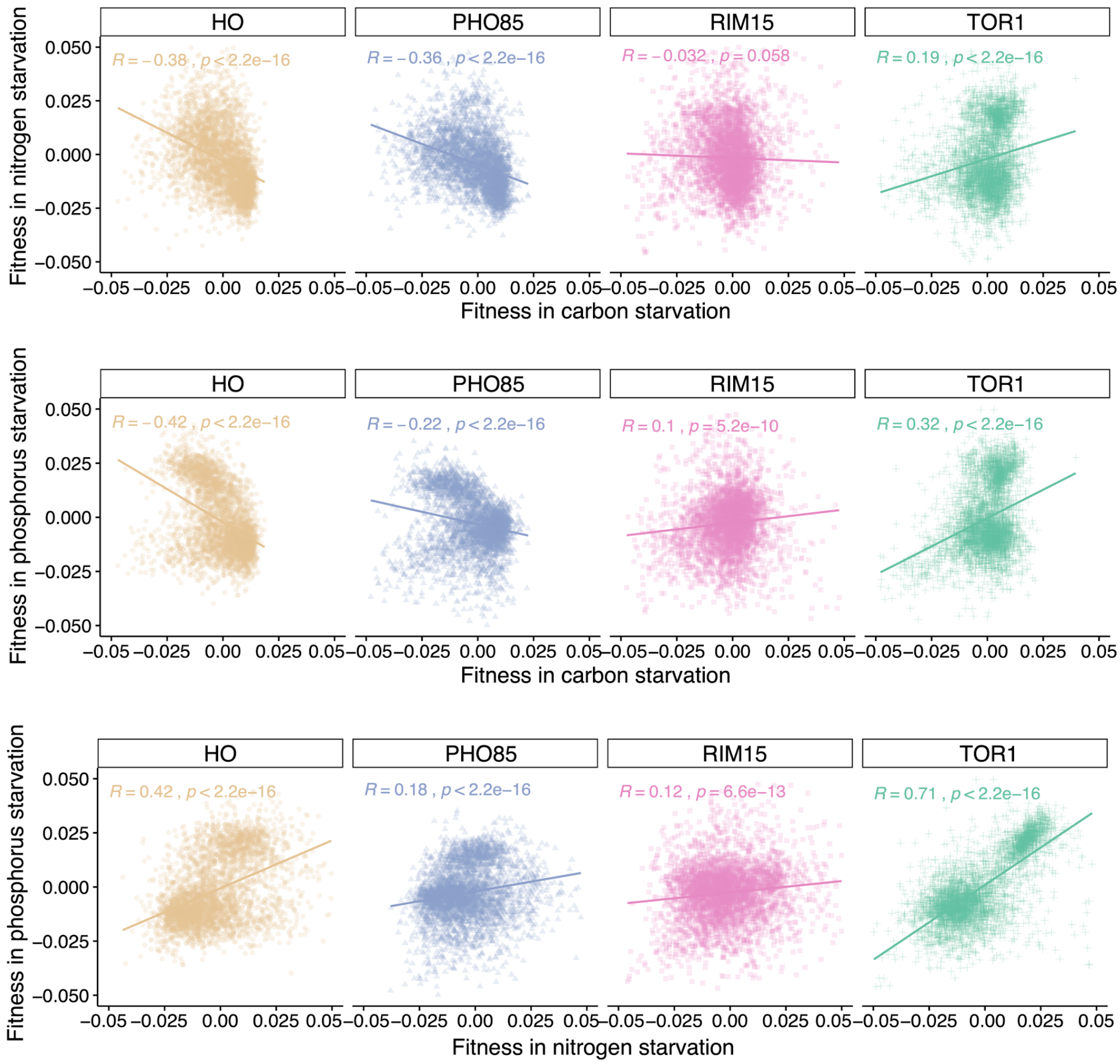

Appendix FigureS1. Correlation of fitness profiles for four mutant libraries across different conditions. Supplementary correlation plot for Figure 1B.

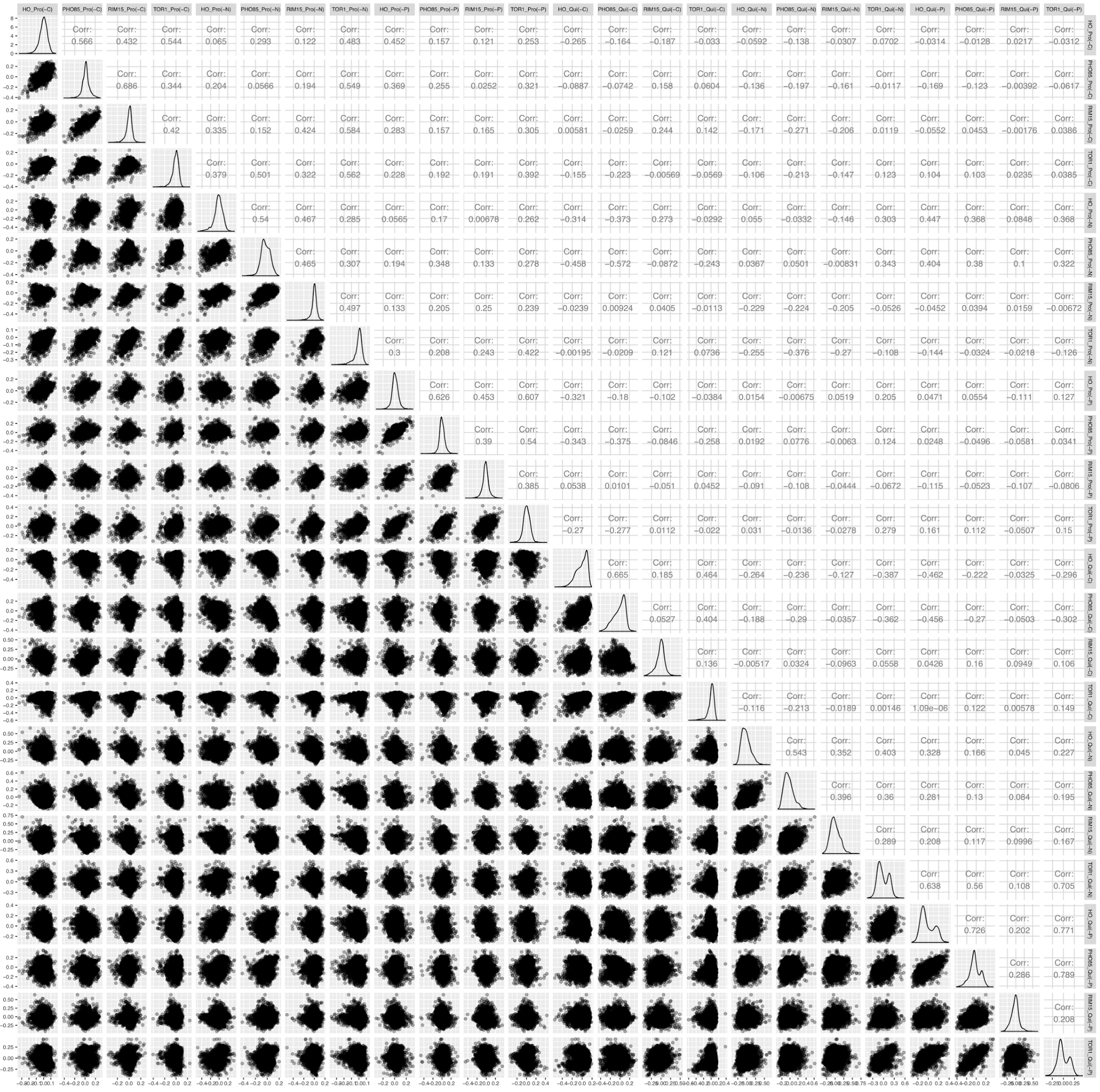

Appendix FigureS2. Pairwise correlation matrix of four mutant libraries across all conditions and cellular states (source data for Fig 1E). Order of matrix (left->right, top->bottom): HO_Pro(-C), PHO85_Pro(-C), RIM15_Pro(-C), TOR_Pro(-C), HO_Pro(-N), PHO85_Pro(-N), RIM15_Pro(-N), TOR_Pro(-N), HO_Pro(-P), PHO85_Pro(-P), RIM15_Pro(-P), TOR_Pro(-P). Label code: Pro - proliferation, Qui - quiescence, -C - carbon restriction, -N - nitrogen restriction, and -P - phosphorous restriction.

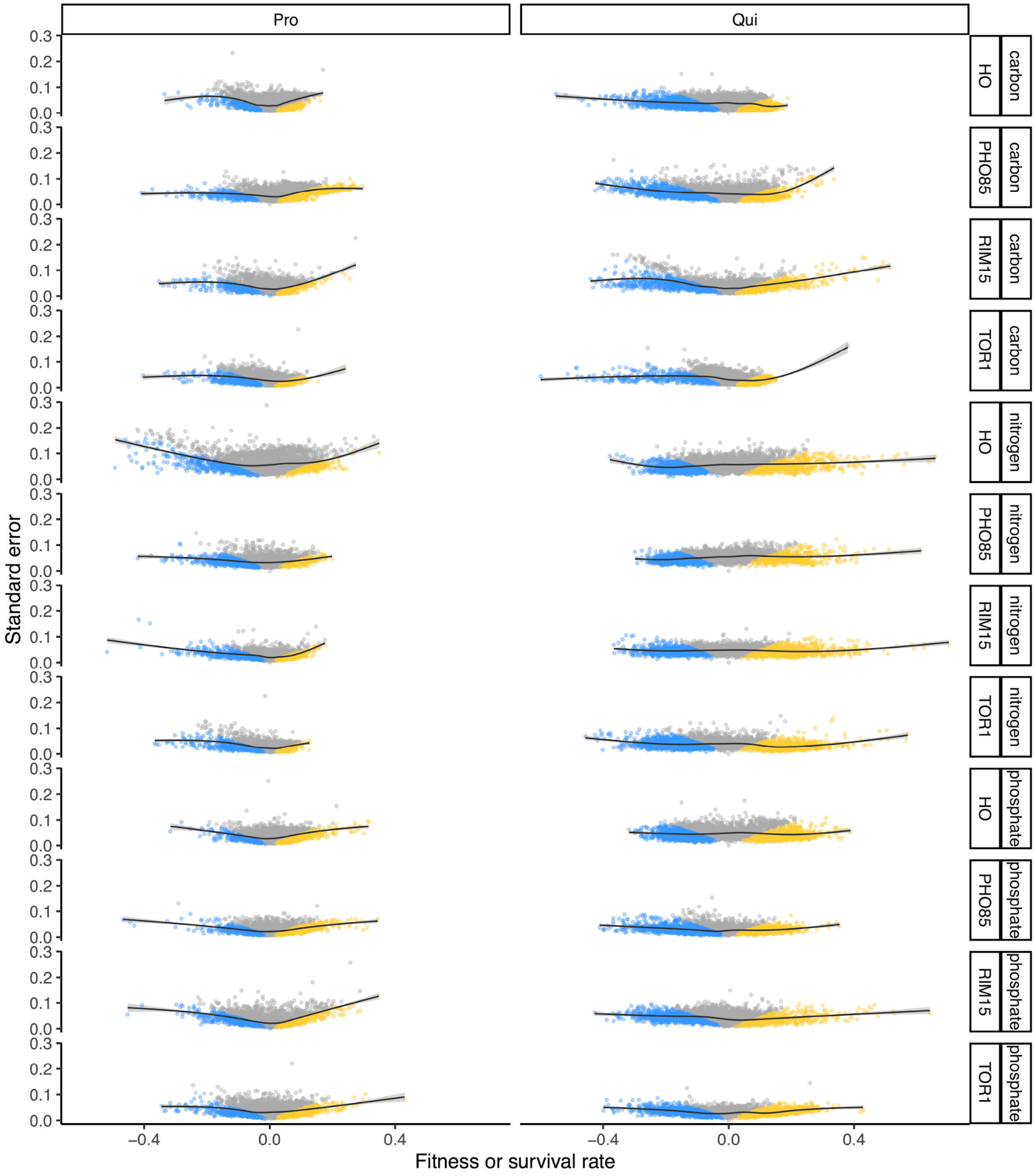

Appendix FigureS3. Diagnostic plots of data quality and model fitting. Scatter plots of the standard error and coefficient for each fitted model (fitness in proliferation and survival in quiescence). The blue and yellow dots are models that are statistically significant with FDR (5%) corrected p-value smaller than 0.05, representing fitness or survival rates that are significantly worse (blue) or better (yellow) in comparison to wild type. Label code: Pro- proliferation, Qui- quiescence.

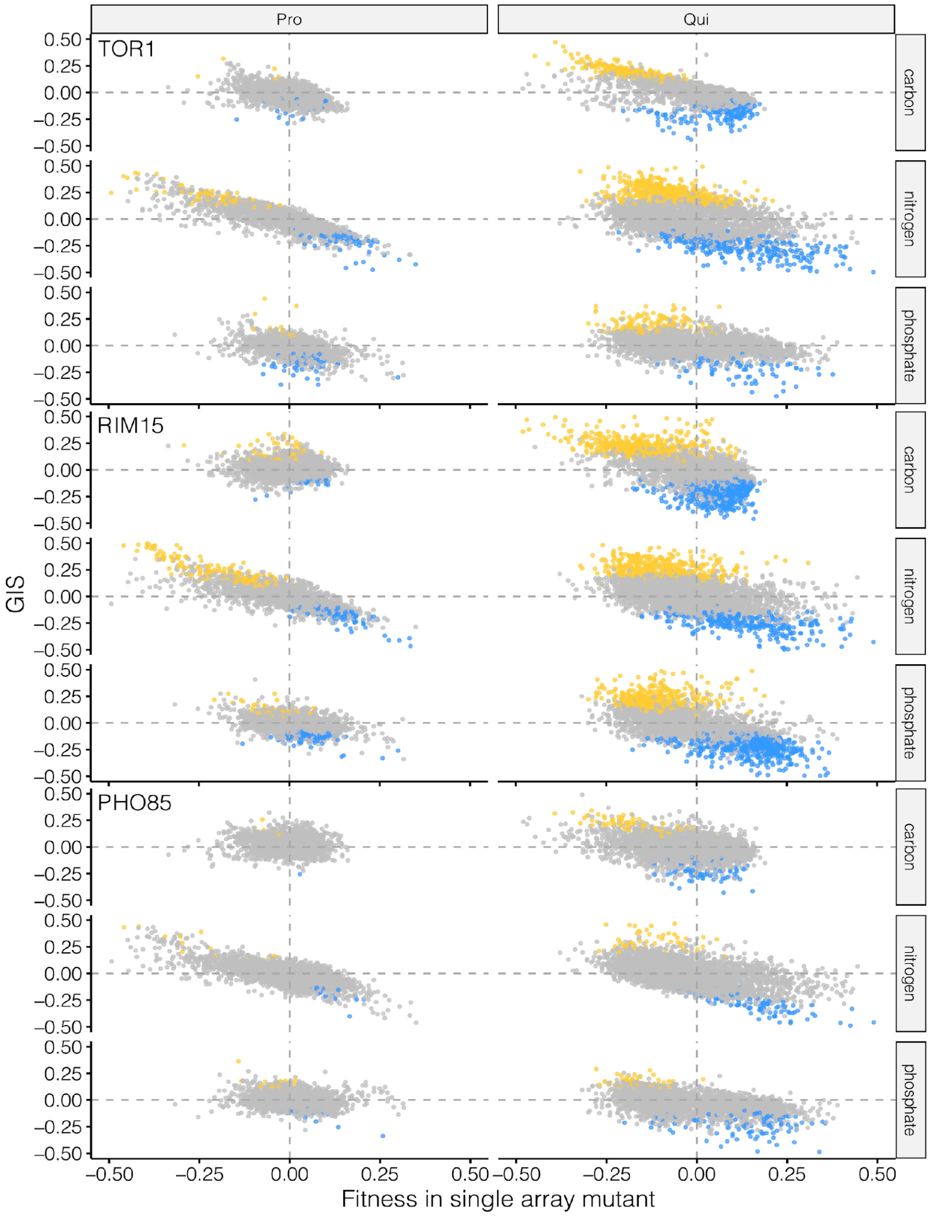

Appendix FigureS4. Comparison of GIS estimated by ANCOVA and single mutant fitness in response to different nutrient restrictions and cellular states. The blue and yellow dots are genetic interactions that are statistically significant with FDR (5%) corrected p-value smaller than 0.05, representing the significant positive (yellow) or negative (blue) with each kinase in labeled condition. Label code: Pro- proliferation, Qui- quiescence.

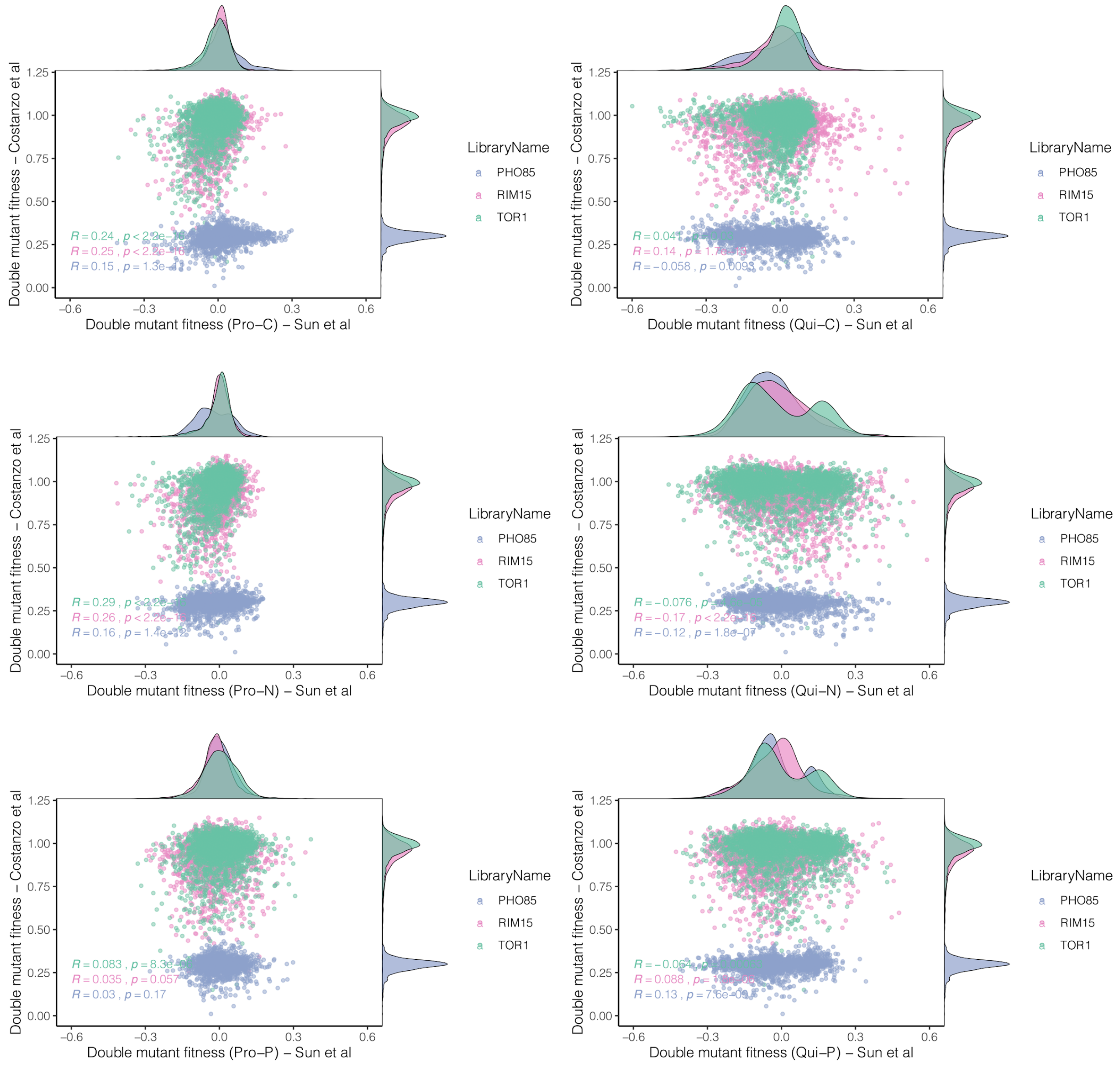

Appendix FigureS5. Double mutant fitness and survival profiles comparison with the data from Costanzo et al's study. Comparison of double mutant fitness/survival profiles between our study and Costanzo et al's study for all three kinases. Pearson correlation score is labeled in the plot with p-value. Label code: Pro - proliferation, Qui - quiescence, -C - carbon restriction, -N - nitrogen restriction, and -P - phosphorus restriction.

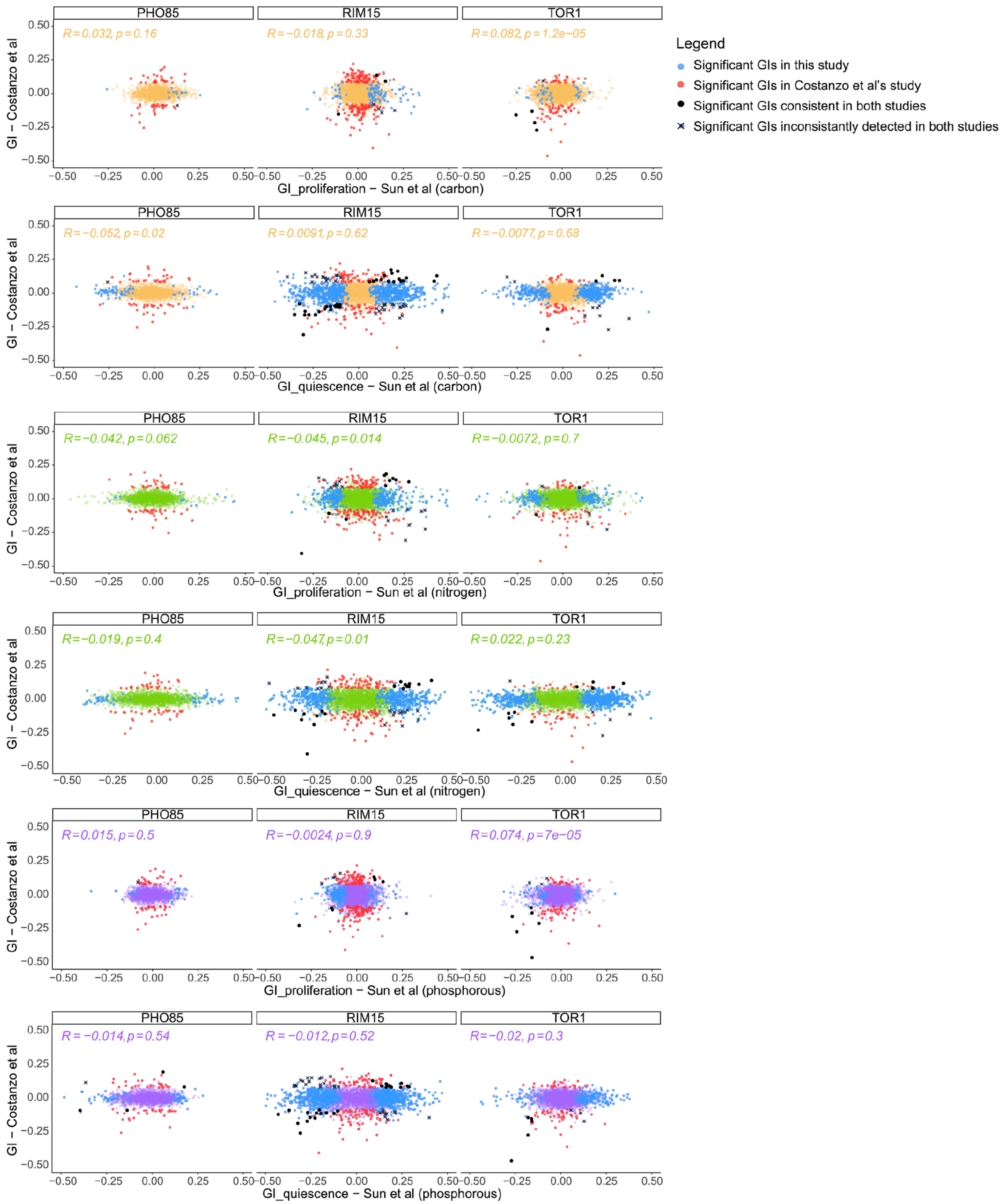
Legend
Significant GIs in this study
Significant GIs in Costanzo et al's study
Significant GIs consistent in both studies
Significant GIs inconsistantly detected in both studies
PHO85
RIM15
TOR1
R=0.032, p=0.16
R=−0.018, p=0.33
R=0.082, p=1.2e−05
GI_proliferation − Sun et al (carbon)
R=−0.052, p=0.02
R=0.0091, p=0.62
R=−0.0077, p=0.68
GI_quiescence − Sun et al (carbon)
R=−0.042, p=0.062
R=−0.045, p=0.014
R=−0.0072, p=0.7
GI_proliferation − Sun et al (nitrogen)
R=−0.019, p=0.4
R=−0.047, p=0.01
R=0.022, p=0.23
GI_quiescence − Sun et al (nitrogen)
R=0.015, p=0.5
R=−0.0024, p=0.9
R=0.074, p=7e−05
GI_proliferation − Sun et al (phosphorous)
R=−0.014, p=0.54
R=−0.012, p=0.52
R=−0.02, p=0.3
GI_quiescence − Sun et al (phosphorous)
GI − Costanzo et al

Appendix FigureS6. Comparison of genetic interactions between data from our study and Costanzo et al's study. Comparison of genetic interaction profiles between our study and Costanzo et al's study for all three kinases. Pearson correlation score is labeled in the plot with p-value (color code: yellow - carbon limitation, green - nitrogen limitation, purple - phosphorus limitation, blue - significant GIs in our study ($p.adj < 0.05$), red - significant GIs in Costanzo et al's study($p.adj < 0.05$), black dot - Significant GIs consistent in both studies, black cross - significant GIs inconsistently detected in both studies).

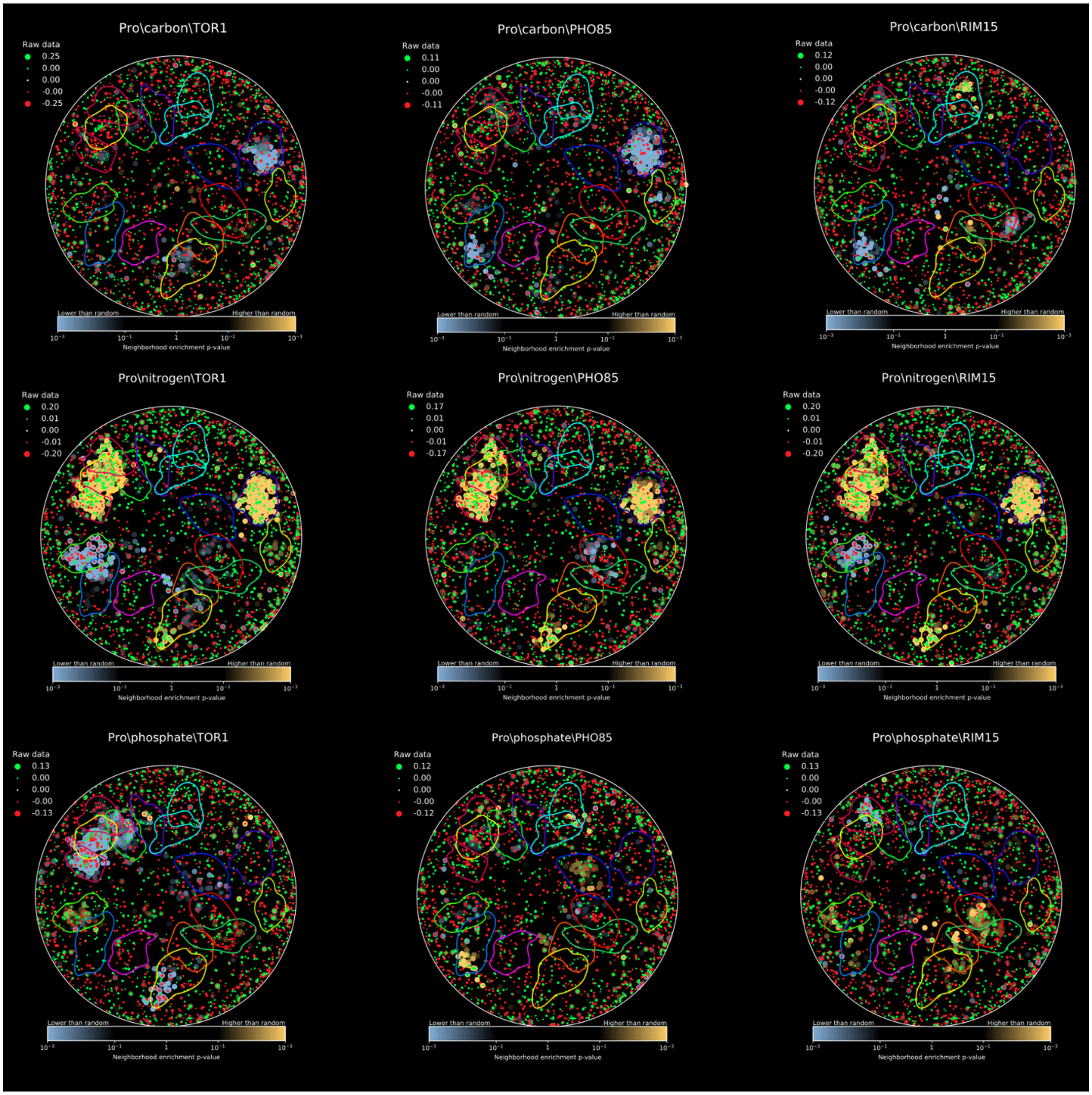

Appendix FigureS7. SAFE analysis of genetic interaction profiles in proliferating cells with raw data. Only the genes with a value are plotted as green or red. Green dots represent positive interactions and red dots indicate negative interactions. The size of each green or red dots is associated with the absolute value of interaction strength. Note that the scale of each plot is different.
