# Supplementary material for: Genetic interaction profiles of regulatory kinases differ between environmental conditions and cellular states
Source: Mol Syst Biol. 2020 May 25;16(5):e9167. doi: 10.15252/msb.20199167 (PMC7247079; doi:10.15252/msb.20199167)
Supplement: Supplementary file 2 — Expanded View Figures PDF [file MSB-16-e9167-s002.pdf]

Expanded View Figures

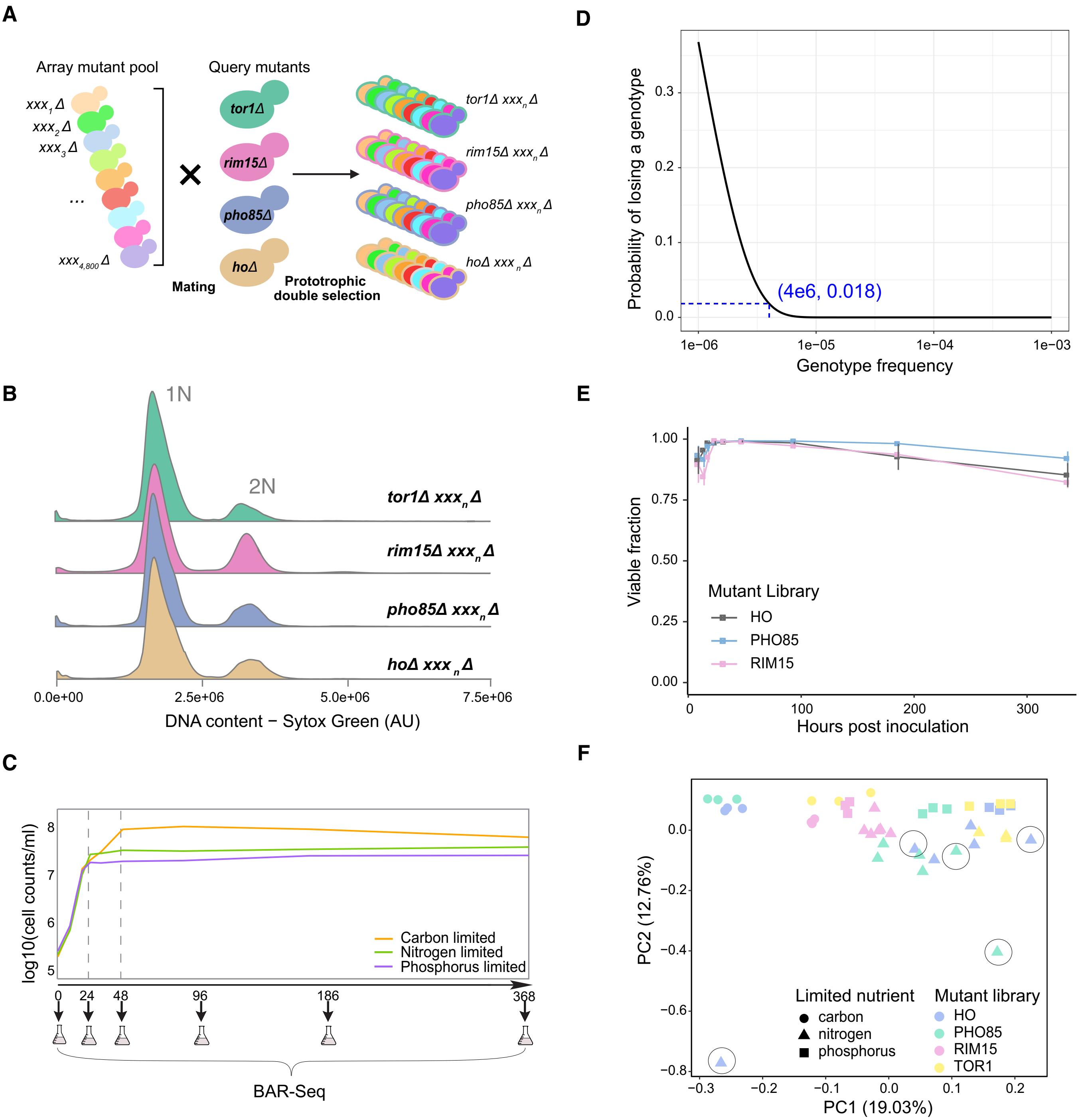

Figure EV1.

Figure EV1. Ploidy confirmation, growth tracking, and data cleaning.

A Genome-wide double mutant prototrophic library construction using synthetic genetic array (SGA). The yeast deletion collection (*xxx*$_n$*Δ::natMX*) was mated with query strains deleted for one of three genes that encode regulatory kinases important in quiescence: *TOR1 (tor1Δ::kanMX)*, *RIM15 (rim15Δ::kanMX)*, and *PHO85 (pho85Δ::kanMX)*. A control library was made by mating the deletion collection to a neutral gene deletion of *HO* (*hoΔ::kanMX*).
B Ploidy confirmation for double mutant libraries assayed using SYTO Green staining.
C Growth curve of 12 mutant libraries, 4 different mutant libraries (*HO*, *RIM15*, *TOR1*, and *PHO85*) × 3 replicates (mean), in different media (orange—carbon restriction, green—nitrogen restriction, and purple—phosphorus restriction).
D Probability of missing a genotype with given frequency. The probability is estimated based on binomial distribution.
E Population viability for mutant library *HO*, *PHO85*, and *RIM15*. Viable fraction was quantified by PI/Syto9 staining followed by flow cytometry.
F PCA for identifying poorly correlated replicates. Circled libraries have been removed for later analysis.

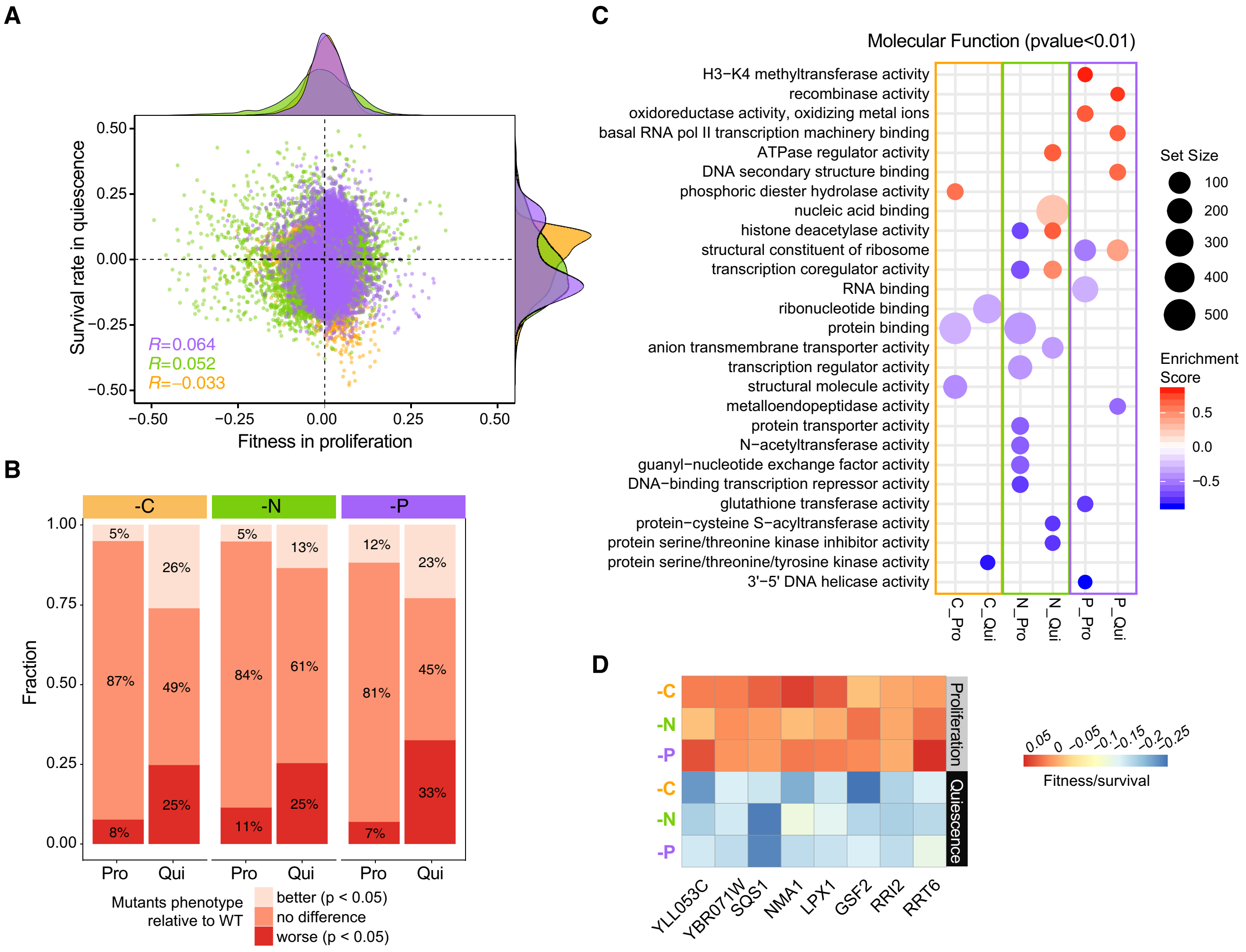

Figure EV2.

 © 2020 The Authors

◀

Figure EV2. Correlation between single mutant (*HO*) fitness in proliferation and survival in quiescence.

A Weak correlation between fitness in proliferation and survival in quiescence under three nutrient-restricted conditions. The distributions of fitness and survival for thousands of mutants in different conditions are shown on the top (fitness) and right (survival). The Pearson correlation score is on the bottom left of the plot with $P < 0.05$. All scatter plots, frequency plots, and labels are colored based on media types (orange—carbon restriction, green—nitrogen restriction, and purple—phosphorus restriction).

B The proportion of mutants with different fitness and survival compared to wild type in proliferation (Pro) and quiescence (Qui) across three nutrient restrictions. Proportions were calculated based on the statistics summarized from linear regression modeling. Better than wild type—regression coefficients—of those mutants are larger than 0 with corrected $P < 0.05$; no difference compared to wild type are the mutants with corrected *P*-value of regression coefficient > 0.05; worse than wild type are the mutants whose regression coefficient is < 0 with corrected $P < 0.05$.

C Enriched GO terms identified using Gene Set Enrichment Analysis (GSEA). GSEA was applied to a ranked gene list based on the fitness for proliferative cells and survival for quiescent cells estimated by linear regression modeling. The false discovery rate (FDR) was set at 0.05. Positive enrichment scores (red) indicate functions that have increased fitness or survival relative to WT control. Negative enrichment scores (blue) indicate functions that when impaired result in decreased fitness or survival. Set size indicates the gene number in each enriched term.

D The 8 genes defined in Fig 2C and their corresponding phenotypic readout in different conditions and cellular states.

© 2020 The Authors

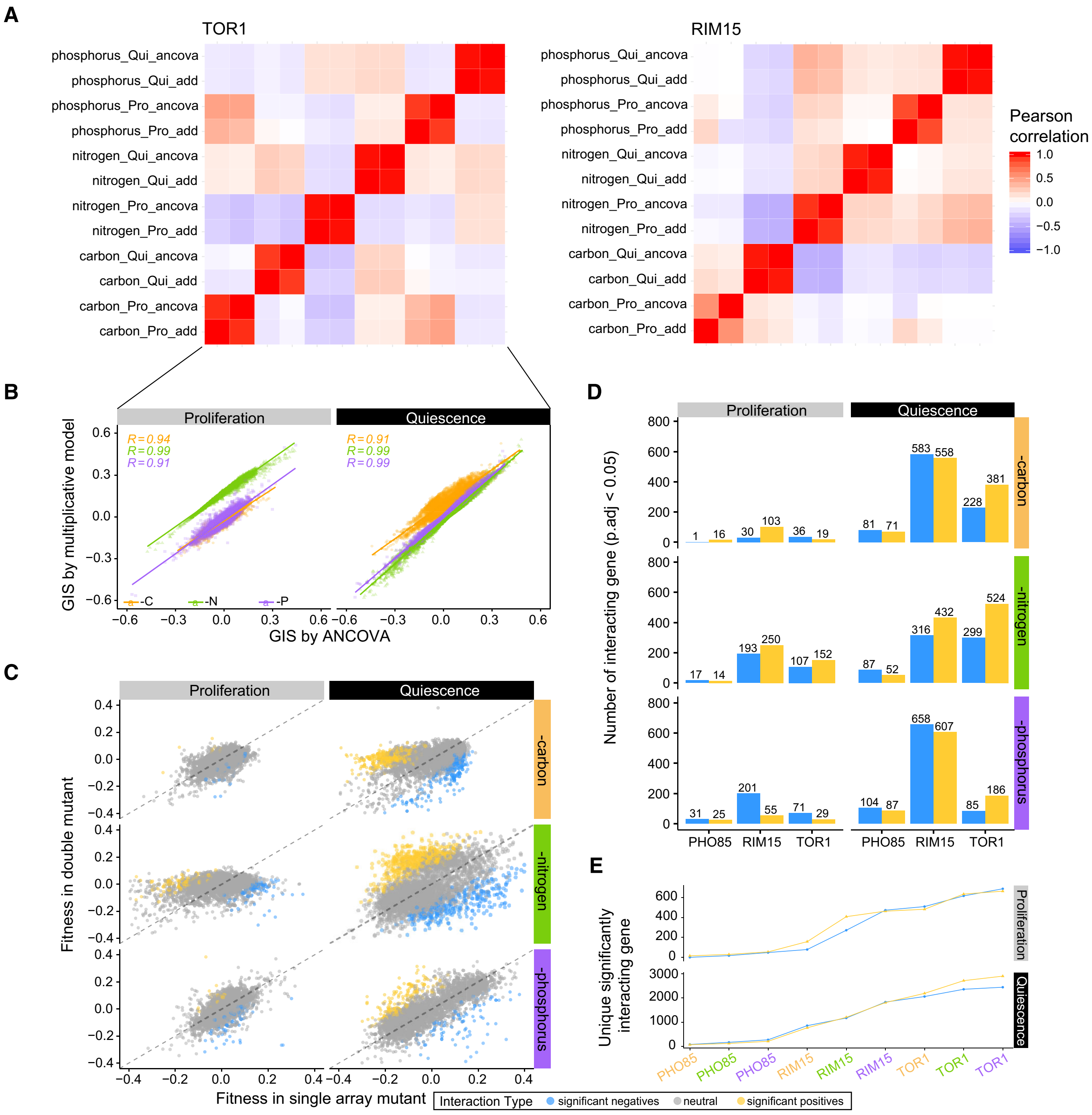

Figure EV3. Genetic interaction quantification comparison and summary.

A Comparison of genetic interaction profiles for kinase gene *TOR1* (left panel) and *RIM15* (right panel) between different quantification models in all conditions.

B Comparison of genetic interaction score between kinase gene *TOR1* and other non-essential genes estimated by ANCOVA and multiplicative model for different cellular states. The Pearson correlation between ANCOVA and multiplicative model calculated GIS is labeled on the top left of the plot with $P < 0.05$. A linear regression line is plotted for each condition for each cellular state. Both scatter plot, linear regression line, and labels are colored based on media types (orange—carbon restriction, green—nitrogen restriction, and purple—phosphorus restriction).

C Scatter plot of fitness and survival estimated in double mutation background (*tor1Δ0 xxx*$_n$*Δ0*: *y*-axis) and single mutation background (*xxx*$_n$*Δ0*: *x*-axis). The dashed diagonal line is colored gray.

D Quantitative summary of significantly interacting genes ($P < 0.05$) with each kinase in proliferation and quiescence.

E Cumulative plot of unique genetic interactions detected with each kinase in three nutrient media (orange—carbon restriction, green—nitrogen restriction, and purple—phosphorus restriction).

 © 2020 The Authors

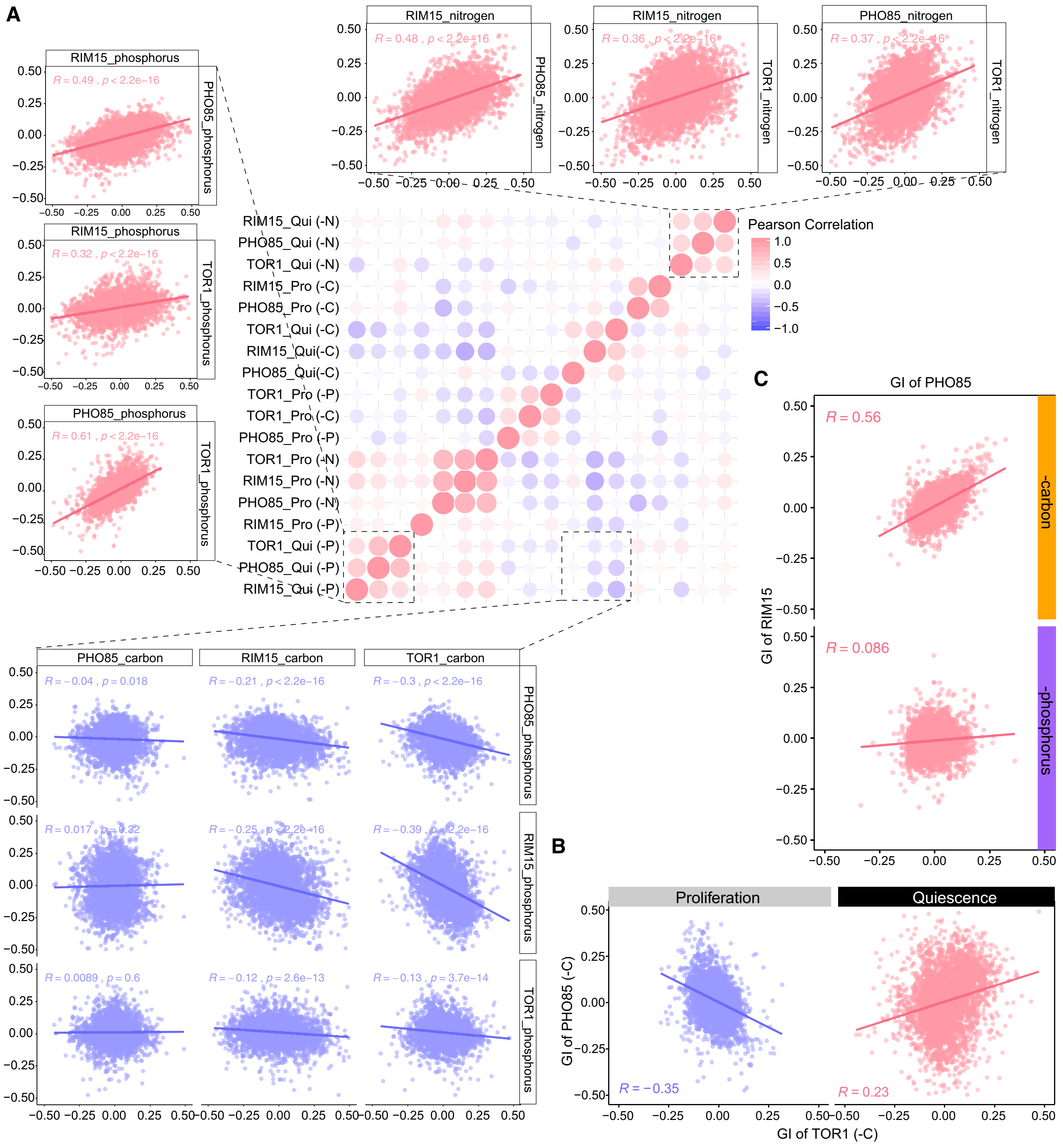

Figure EV4. Genetic interaction profiles with kinases across different conditions.

A Correlation heatmap of genetic interaction profiles for each kinase under two cellular states in response to different nutritional restrictions. Samples are orders based on hierarchical clustering. The Pearson correlation score is labeled in the scatter plot with *P*-value (color code: pink—positive correlation, purple—negative or no significant correlation).

B Comparison of genetic interaction profiles of *TOR1* and *PHO85* between carbon-restricted proliferative and carbon-starved quiescent cells. Calculated Pearson correlation coefficient is plotted on the bottom of each panel with $P < 0.05$ (color code: pink—positive correlation, purple—negative correlation).

C Comparison of genetic interaction profiles between *PHO85* and *RIM15* in carbon-restricted (top) or phosphorus-restricted (bottom) proliferating cells. The Pearson correlation score is labeled in the plot with $P < 0.05$ (color code: pink—positive correlation).

© 2020 The Authors

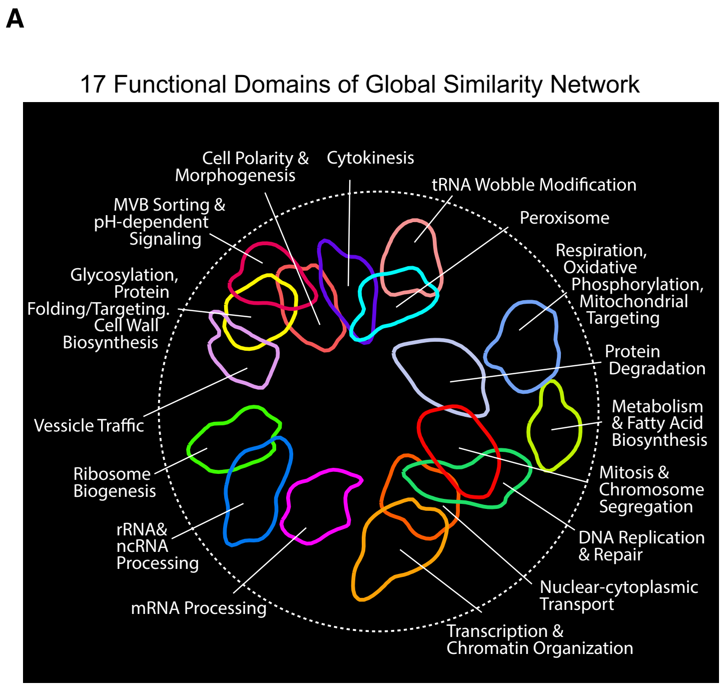

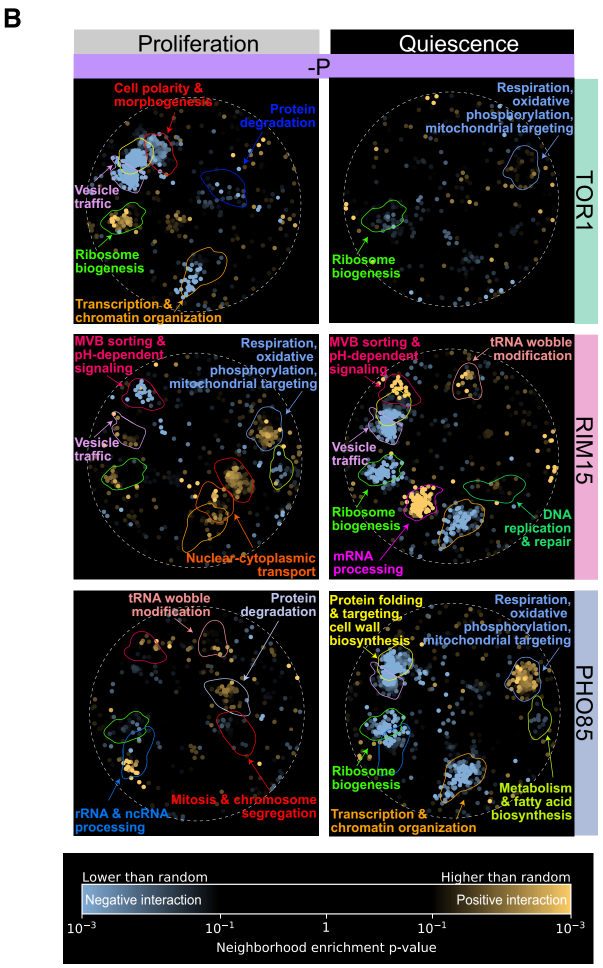

Figure EV5. A global network of genetic interaction profile similarities.

A A global genetic profile similarity network encompassing ~3,971 non-essential and essential genes from Costanzo *et al* (2016). The global similarity network was annotated using the Spatial Analysis of Functional Enrichment (SAFE) (Baryshnikova, 2016), identifying network regions enriched for similar GO biological process terms, which are color-coded (Costanzo *et al*, 2016).

B Genetic interaction enrichment landscape of *TOR1, RIM15, and PHO85* in proliferating and quiescent cells under phosphorus (-P) restriction. Each dot represents one gene. Blue dots represent genes that have negative interactions with corresponding kinase (row-wise) in each condition (column-wise), and yellow dots represent genes with positive interactions.

 © 2020 The Authors

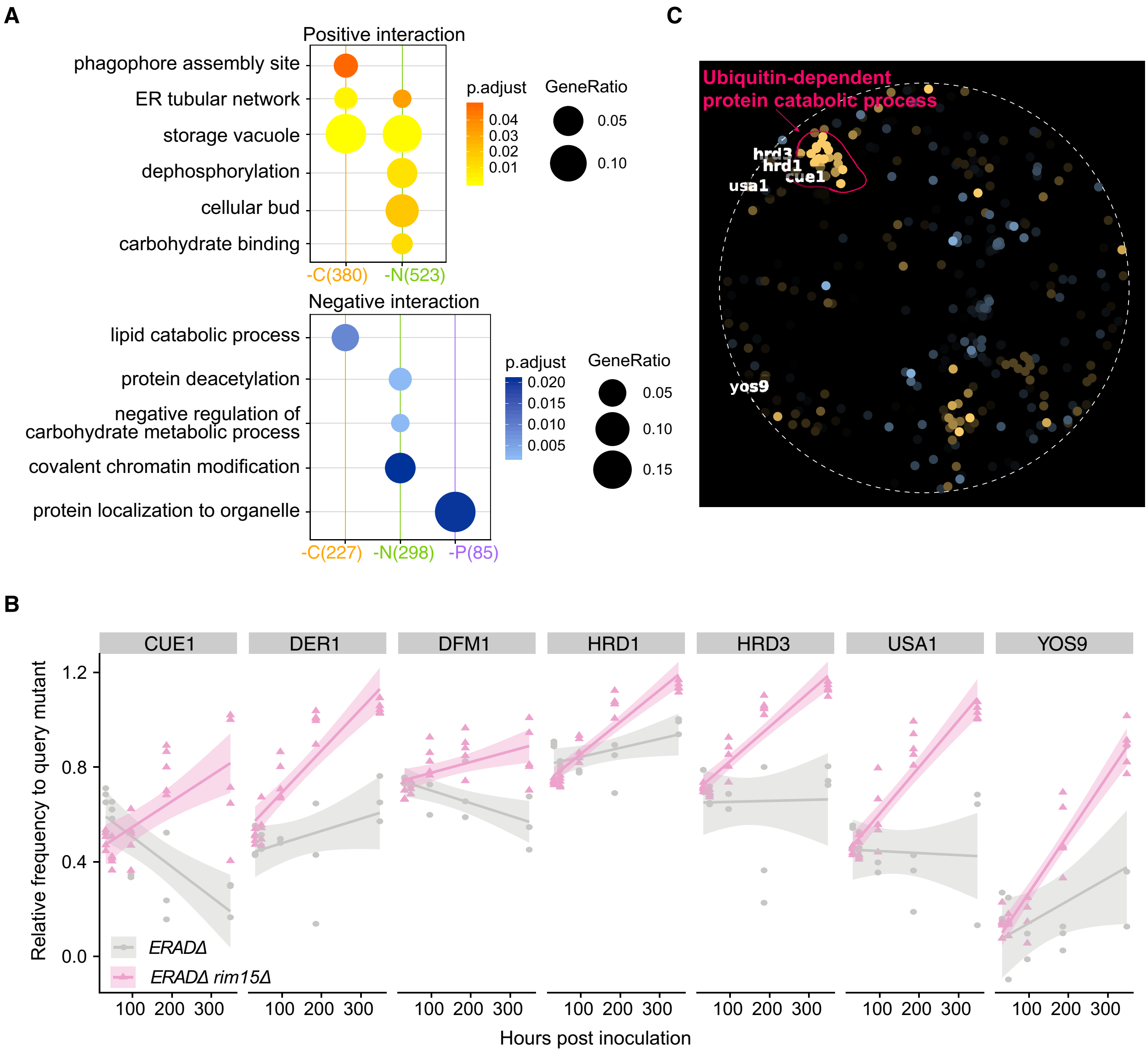

Figure EV6. Functional analysis of significantly interacting genes with different kinases.

A GO term enrichment analysis for genes that significantly interact with *TOR1* in each different nutrient starvation condition. Only GO terms with significant representation are shown ($P_{adj} < 0.05$). The same color schemes are used to represent different interaction types (yellow—positive, blue—negative). The intensity of the dot color represents the significance; for example, the lighter the color is, the smaller the *P*-value is. The size of the dot represents the gene group size within each term, given the significant interacting genes under each condition (colored parentheses on *x*-axis).

B Relative frequency of each double (*ERADΔ0 rim15Δ0*) and single mutant (*ERADΔ0*) as a function of time in response to nitrogen starvation.

C SAFE analysis for genes interacting with *RIM15* in nitrogen starvation conditions.

© 2020 The Authors
